# Comparing the Fracture Resistance and Modes of Failure in Different Types of CAD/CAM Zirconia Abutments with Internal Hexagonal Implants: An In Vitro Study

**DOI:** 10.3390/ma15072656

**Published:** 2022-04-04

**Authors:** Yu-Tsen Chang, Yu-Ling Wu, Hung-Shyong Chen, Ming-Hsu Tsai, Chia-Chen Chang, Aaron Yu-Jen Wu

**Affiliations:** 1 Department of Dentistry, Kaohsiung Chang Gung Memorial Hospital and Chang Gung University College of Medicine, Kaohsiung 833, Taiwan; ash-again@hotmail.com (Y.-T.C.); oxalis76@cgmh.org.tw (Y.-L.W.); dentalbear2001@yahoo.com.tw (C.-C.C.); 2 Department of Mechanical Engineering, Cheng Shiu University, Kaohsiung 833, Taiwan; hschen@gcloud.csu.edu.tw (H.-S.C.); k0635@gcloud.csu.edu.tw (M.-H.T.)

**Keywords:** fracture resistance, modes of failure, zirconia abutment, titanium insert

## Abstract

Three groups of zirconia abutments (*n* = 5) consisting of different connection designs or manufacturers were investigated (All-Zr, ASC-Zr, and AM-Zr groups). All-electric dynamic test instruments were used to place static loading on a specimen with a crosshead speed set at 1 mm/min. A Kruskal–Wallis test and a post hoc Mann–Whitney U test were used for statistical evaluation. The mean fracture resistance was 252.37 ± 82.79 N for the All-Zr group, 384.62 ± 45.24 N for ASC-Zr group, and 361.83 ± 90.31 N for the AM-Zr group. The difference of fracture resistance between the three groups was marginally significant (Kruskal–Wallis test, *p* = 0.054), with the ASC zirconia abutment tending to have higher fracture resistance than the full zirconia abutment. The modes of failure among the three types of abutments are different. The All-Zr group showed an oblique fracture line starting from the buccal aspect at the region of the implant platform. While the ASC-Zr and AM-Zr groups showed a relatively horizontal fracture line with a greater distance from the implant platform. The titanium inserts cannot significantly improve the fracture resistance of the zirconia abutment. However, they may alter the modes of failure, allowing buccal fracture surfaces of the zirconia abutments to be placed away from the implant platform, thereby protecting the implant–abutment connection.

## 1. Introduction

Since their invention, dental implants have been widely applied in dental restorations to restore the occlusal function of patients. The titanium implant abutment has exhibited satisfactory biocompatibility and mechanical properties, making it suitable for long-term use in the implant-supported prosthesis. However, despite the stable and predictable characteristics of titanium abutments, long-term observations from clinical studies had revealed that an unnatural bluish color may, in certain situations, appear in the gingiva surrounding the titanium abutments, particularly when the implant was inserted too labially and when the gingival biotype of the patient is thinner [1,2,3,4,5].

With the development of ceramic materials, computer-aided design and computer-aided manufacturing (CAD/CAM) technology, and patient demands for aesthetically pleasing prostheses, ceramic materials have been gradually applied to implant abutments [6,7]. The first all-ceramic abutment was manufactured by using densely sintered alumina (Al_2_O_3_). However, studies have reported that Al_2_O_3_ has low fracture resistance [8,9]. Zirconia was used as abutment material in 1996, as it is more resistant to fracture than alumina [10]. Zirconia allows for a certain degree of light transmission, enabling dental technologists to create prostheses that satisfy patients’ aesthetic needs [11].

Zirconia abutments can be manufactured in the form of one-piece or two-piece abutments. One-piece abutments are made entirely of zirconia in their abutment body and implant–abutment connections. In vitro studies have reported that these abutments are sufficiently resistant to the maximal incisal force in the anterior region (90–370 N) [12,13,14,15,16]. Mitsias et al. [17] reported that the mean fracture resistance of one-piece zirconia abutments with internal connection is 690 ± 430 N. Kim et al. [18] tested one-piece CAD/CAM zirconia abutments with internal connection, using static loading, and reported a mean fracture load of 480.01 ± 174.46 N. However, numerous clinical reports have recorded cases where one-piece zirconia abutments fractured [19]. Leutert et al. [20] compared titanium and one-piece zirconia abutments based on their fracture resistance under static load. The one-piece zirconia abutments showed a low fracture strength when attached to tissue-level Straumann implants (158 ± 34.7 N), thus making them inapplicable in clinical use, regardless of the implant’s position. In a randomized controlled trial, Ferrari et al. [21] reported that, during 3 years of clinical observation, 5 out of 28 CAD/CAM one-piece zirconia abutments fractured at the abutment joints. They concluded that this type of zirconia abutment should be limited to the anterior region and avoided in the posterior load-bearing area. These findings led to the invention of two-piece abutments comprising titanium inserts and a zirconia abutment bodies and can be attached through friction-fit or bonding manner. These abutments are reported to be both aesthetically pleasing and strongly resistant to fracture [22]. In a retrospective clinical study, Lin et al. [23] concluded that the two-piece zirconia abutment appears to be a suitable treatment option for the anterior and premolar regions.

In clinical reality, dentists, patients, and dental technologists may opt for non-original abutments, due to their lower cost and convenience [24]. Some aftermarket manufacturers have begun producing CAD/CAM zirconia abutments; several non-original prosthetic kits are also available on the market. However, standards and regulations have not been formulated for the design and quality of manufactured abutments. Limited studies have compared the mechanical properties of original two-piece equipment manufacturing (OEM) and aftermarket zirconia abutments. The effects of the design changes on implant–abutment connection on mechanical properties and intraoral performance of the entire implant system are unclear [25]. Further research is needed to verify whether the zirconia abutment fabricated by aftermarket manufacturers has the mechanical properties required for clinical use.

Our in vitro study aimed to compare fracture resistance and modes of failure under static loading in three different types of zirconia abutments attached to implants with standard diameters and internal hexagonal connections. Our proposed null hypothesis states that these zirconia abutments do not differ in fracture resistance and failure modes under static loading.

## 2. Materials and Methods

**Figure 1:**
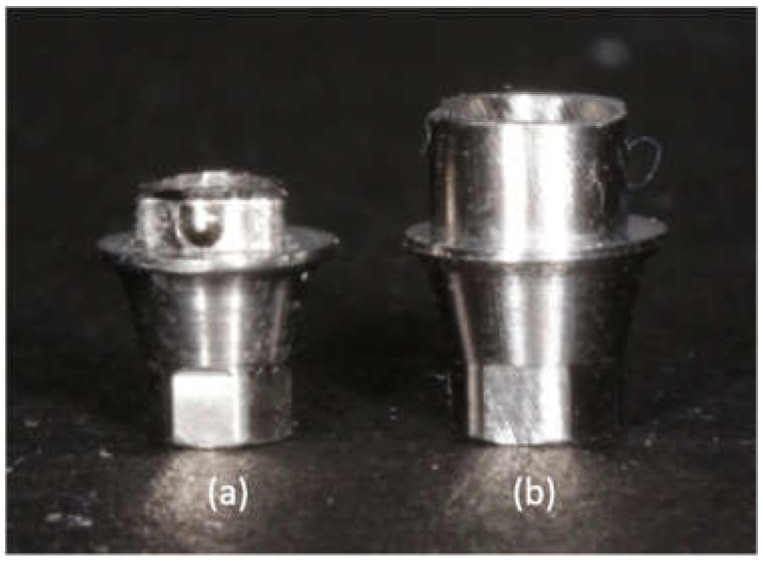
Titanium insert of NobelProcera ASC abutment (**a**) and aftermarket abutments (**b**).

Nine zirconia implant abutments with the following three designs (*n* = 5) were used (Table 1): The abutments of group All-Zr are one-piece implant abutments, which are entirely composed of zirconia (NobelProcera CAD/CAM Zirconia Abutment, Nobel Biocare, Yorba Linda, CA, USA). The abutments of ASC-Zr and AM-Zr groups are two-piece implant abutments (Figure 1). The ASC-Zr abutment contains a titanium insert with an axial wall height of 1 mm, which is friction-fitted with the zirconia component (NobelProcera ASC Abutment; Nobel Biocare, Yorba Linda, CA, USA). The AM-Zr abutment contains a 2 mm–high titanium insert fabricated by an aftermarket manufacturer bonded to the zirconia component.

**Figure 2:**
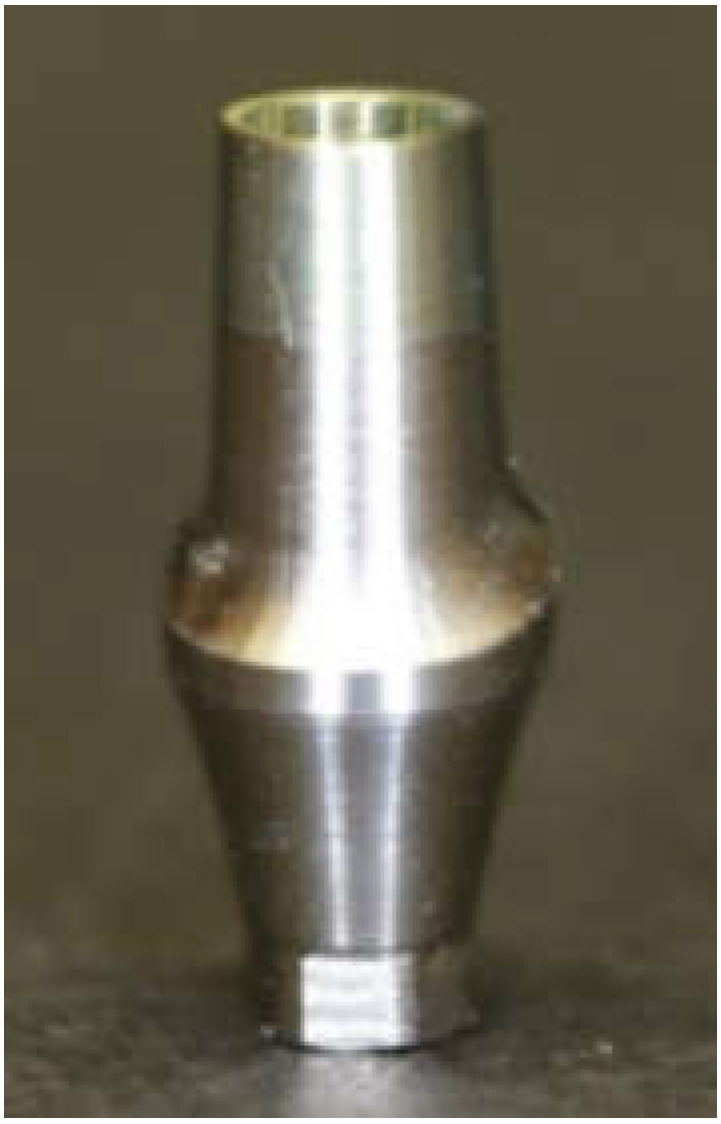
Titanium prototype abutment (NobelReplace Abutment Conical Connection RP 3 mm).

**Figure 3:**
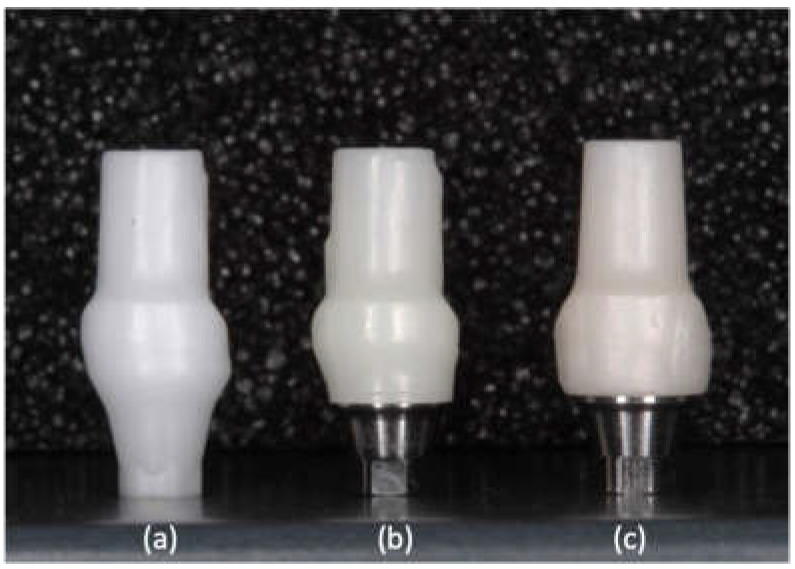
Three groups of zirconia abutments used: All-Zr (**a**), ASC-Zr (**b**), and AM-Zr abutments (**c**).

A prefabricated titanium abutment (NobelReplace Abutment Conical Connection RP, Yorba Linda, CA, USA, 3 mm) (Figure 2) was designated as the prototype abutment and scanned with a scanner. The abutments of All-Zr and ASC-Zr groups were then obtained from OEM manufacturer Nobel Biocare, Yorba Linda, CA, USA, and abutments from the AM-Zr group were milled by the milling machines. In this way, all the abutments are manufactured with identical external dimensions, with a 0.5 mm–deep circumferential chamfer and an incisogingival height of 9.5 mm (Figure 3).

The zirconia abutments of group AM-Zr requires the bonding of titanium inserts. The surface of the titanium inserts and the intaglio surface of the zirconia abutments were sandblasted, and an alloy primer was applied over the titanium insert. The titanium inserts and the zirconia abutments were bonded with dual-polymerized composite resin adhesive bonding. Excess adhesive was removed at ×10 magnification.

Nine implant fixtures (NobelReplace Conical Connection PMC RP 4.3 mm × 10 mm, Gothenburg, Sweden) with an internal hexagonal connection and regular platform were used. By scanning each abutment with a scanner (NobelProcera 2G scanner, Nobel Biocare, Kloten, Switzerland), zirconia crowns with an 11 mm incisogingival height and 8.5 mm mesiodistal width were created for each abutment to simulate the morphology of the central incisor. The implant abutment and the intaglio surface of the zirconia crown were sandblasted and treated with a ceramic primer. A dual-cure resin cement was used to bond the crown to the abutment. After removing the excess adhesive, a preload of 35 N·cm was applied according to the manufacturer’s instructions to tighten all the abutments to the implant fixtures.

**Figure 4:**
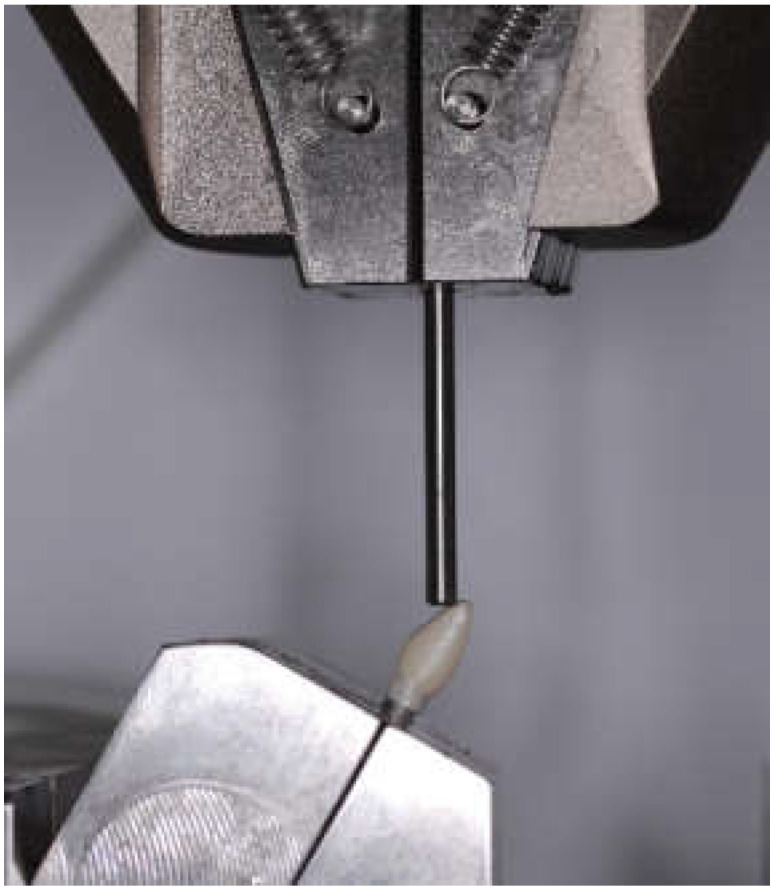
Loading of the assembled specimen with a universal testing machine at 30°.

The specimen was fixed on a metal jig, tilting in a 30° angulation of the long axis of the implant fixture to simulate the Class I incisor relationship [26,27,28,29]. An all-electric dynamic test instrument (ElectroPulsTM E3000, Instron, Norwood, MA, USA) was used to measure the fracture strength of the zirconia abutments (Figure 4). A metal rod was used to place loading at 2 mm lingual from the incisal edge of the zirconia crown. The speed of the testing machine was set at 1 mm/min. The crosshead motion stopped after the load started to decrease due to fracture of the zirconia component or the plastic deformation of the screw or implant fixture. The value was recorded as a failure load, the Kruskal–Wallis test was used for statistical evaluation of the significance of differences between the three groups, and the Mann–Whitney U test was used for post hoc pairwise comparisons. The *p*-values were adjusted by using the Benjamini and Hochberg method. In addition, the failure mode of the abutments was studied and analyzed under a digital microscope (VHX−950F, Keyence, Belgium).

## 3. Results

**Figure 5:**
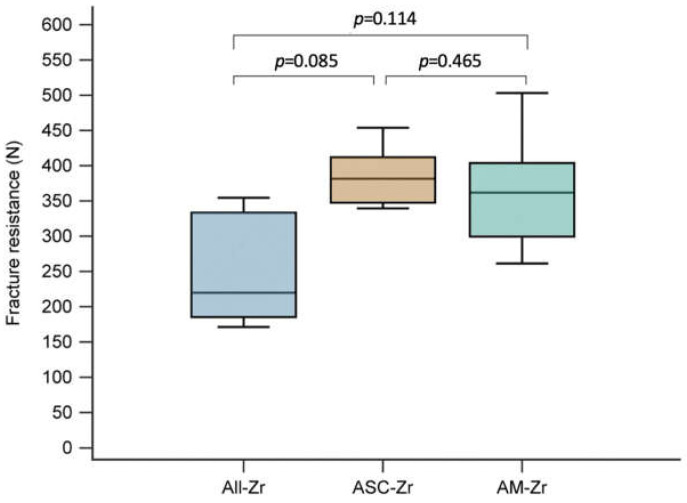
Box plot showing the fracture resistance of ASC-Zr abutments was marginally higher than All-Zr abutments (*p* = 0.085), followed by group-to-group comparisons.

The mean fracture resistance was 252.37 ± 82.79 N for the All-Zr group, 384.62 ± 45.24 N for the ASC-Zr group, and 361.83 ± 90.31 N for the AM-Zr group (Table 2). The difference in fracture resistance between the three groups was marginally significant (Kruskal–Wallis test, *p* = 0.054). The ASC-Zr abutments tend to have higher fracture resistance than full-zirconia abutments (*p* = 0.085; Benjamini and Hochberg method). The differences in fracture resistance between All-Zr and AM-Zr groups and between ASC-Zr and AM-Zr group were not statistically significant (Figure 5).

**Figure 6:**
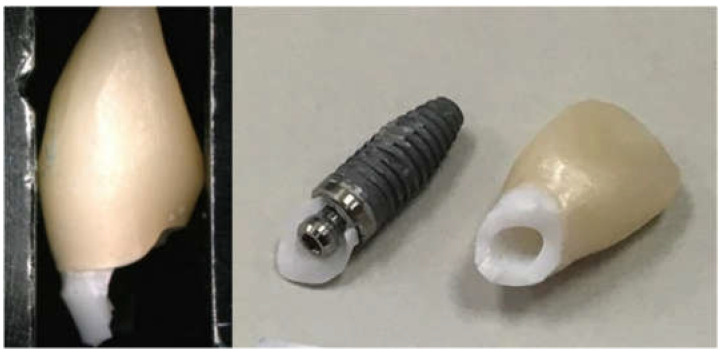
Typical fracture mode in All-Zr group (oblique fracture line starting from the buccal aspect in the internal hexagon region).

**Figure 7:**
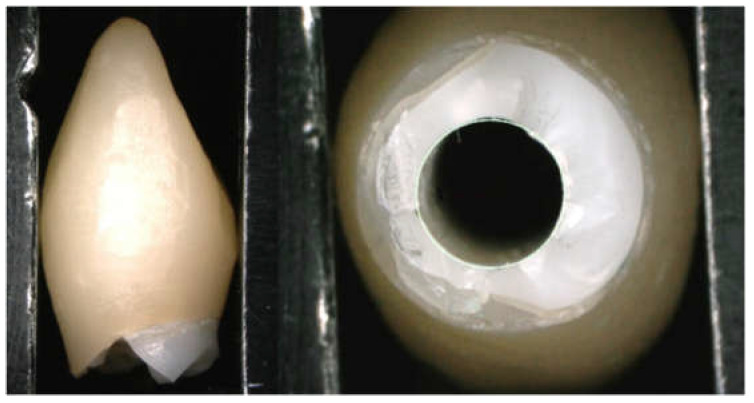
Fracture mode in the ASC-Zr group (a relatively horizontal pattern with a buccal fracture surface away from the implant platform).

**Figure 8:**
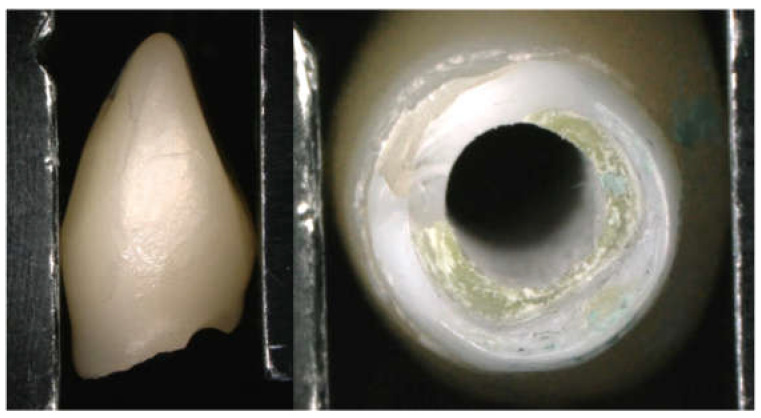
Fracture mode in AM-Zr group (horizontal fracture pattern similar to ASC-Zr group, but the fracture surface is even higher).

The mid-buccal fracture surface’s distance from the platform of the implant fixture was 0 mm for the All-Zr group, 3.52 ± 0.44 mm for the ASC-Zr group, and 4.12 ± 0.13 mm for the AM-Zr group. For the mid-palatal side, it was 5.11 ± 1.47 mm, 3.82 ± 0.74 mm, and 5.18 ± 0.18 mm, respectively (Table 3). There was a significant difference in the buccal and palatal height of the fracture in the All-Zr and AM-Zr groups (*p* < 0.05), but not in the ASC-Zr group (*p* = 0.47). For the distance of the mid-buccal fracture surface from the platform of implant fixture, there was a significant difference (*p* < 0.05) between the three groups; as for the mid-palatal site, there was no significant difference between the three groups (*p* = 0.32). The fracture plane of the abutments presented an oblique pattern in the All-Zr group, whereas the ASC-Zr and AM-Zr groups showed a relatively horizontal fracture plane. The modes of failure among the three types of abutments are different, and all the titanium inserts and screws did not show catastrophic damage or fracture (Figure 6, Figure 7 and Figure 8).

## 4. Discussion

This in vitro study showed that the three different types of zirconia abutments had a comparable fracture resistance but different modes of failure under the static load for the platform-switched internal hexagonal implants. Therefore, the null hypothesis could not be rejected. In recent years, zirconia abutments have been increasingly used in clinical applications for their excellent fracture resistance relative to other ceramic abutments, including abutments made of alumina. Yttrium-stabilized tetragonal zirconia polycrystal (Y-TZP) received the most attention, due to its excellent mechanical properties. However, the impact of the applied load on zirconia abutment is still inconclusive. Studies have determined that the type of implant–abutment connection, the physical properties of raw stock, and the manufacturing and experimental methods may significantly affect the abutment strength [30]. Xu et al. [31] studied the effect of grinding parameters on the strength of Y-TZP. Its strength increased substantially only when a 25 μm diamond wheel was used in fine machining, whereas coarse grinding resulted in decreased strength. The coarser the diamond wheel they used, the lower the measured strength.

Cyclic loading, which simulates fatigue loading, is the cause of most clinical failures. However, static load tests can simulate situations where the implant complex hits a hard object and is traumatized. In some cases, such as patients with parafunction habits (e.g., bruxism, clenching, etc.), the incisive force could also be much higher than the physiological range [32]. Cyclic and static loading are two independent conditions, and both may affect the settling of the implant–abutment connection after occlusal load [33]. Gehrke et al. [22] investigated the fatigue resistance of one- and two-piece CAD/CAM zirconia abutments. They reported that two-piece abutments with an internal-hex connection demonstrated greater fracture resistance than one-piece zirconia abutments and may be clinically beneficial in high-load areas, such as posterior tooth replacements.

The weak point of many all-ceramic abutments is located at the implant–abutment interface. The two-piece abutment design provides metal reinforcement at the implant–abutment connection for greater fracture resistance and possesses the ideal aesthetic of all-ceramic abutment [22]. Stimmelmayr et al. [34] reported that the zirconia implant abutments attached to the titanium core showed higher fracture strength than the one-piece zirconia abutments, indicating that they might be more suitable for clinical use. The authors also suggested that titanium implants exhibited higher interface wear under cyclic loading when attached to one-piece zirconia abutments than attached to titanium abutments [35]. In this regard, a two-piece abutment with titanium–titanium connections may benefit clinical applications.

It has been reported that implant abutments with internal hexagonal connections are more stable than external hexagonal designs, due to their wider stress distribution across the interface [36]. However, Sailer et al. [26] concluded that internally connected one-piece zirconia abutments under oblique loading are less resistant to fracture than externally connected one-piece zirconia abutments. The mean fracture load of externally connected (NobelBiocare, Yorba Linda, CA, USA) zirconia abutments is 480.9 N (±182.8), whereas that of internally connected (Straumann, Basel, Switzerland) zirconia abutments is 292.0 N (±218.4), which is similar to the maximum load measured in the present study. The fracture resistance of the three types of zirconia abutments is within the physiological shear range of the anterior zone (approximately 90 to 370 N). Whether the mechanical strength is sufficient for long-term use in the anterior region remains to be confirmed by more clinical studies.

Along with introducing CAD/CAM and aftermarket prosthetic components into the clinical use, non-original abutments receive more attention. Although aftermarket zirconia abutments showed similar fracture resistance with OEM zirconia abutments in the current study, Gigandet et al. [24] reported that aftermarket abutments that changed the original design and materials showed higher rotational misfit. This mismatch may result in increased wear and micromotion between the titanium and zirconia interfaces, leading to surface flaws and zirconia cracks [37]. By comparing the torque loss of four types of abutments, Park et al. [38] reported that the torque loss of the original abutments was lower than the copy abutments. Similar results were obtained by Alonso-Pérez et al. [39], who concluded that internal precision at the implant–abutment connection is a crucial determinant to the mechanical properties of abutments, possibly explaining the superiority of original abutments over its non-original counterparts. However, few clinical studies have evaluated the effects of non-original abutments on implants. Therefore, more clinical studies should be conducted to test the incidence of failure and complications of implant complexes with the original and non-original connections.

In the All-Zr group, the zirconia abutments showed an oblique fracture pattern. The fracture surface was higher at the palatal side of the abutments and significantly lower at the buccal side, showing an oblique fracture line starting from the buccal aspect at the region of the implant platform. This finding is similar to the study results reported by Nothdruft et al. [28]. The fracture surface showed a relatively horizontal pattern in zirconia abutments with a titanium insert, demonstrating that titanium inserts raise the buccal fracture surface away from the implant platform. The level of fracture surface at the buccal side of the zirconia abutments increased with the height of the titanium inserts, which may protect the implant–abutment connection. Kim et al. [40] compared the failure modes among three different zirconia abutments after static load. The abutment consisted entirely of zirconia, which showed fractures arising from the connection area. The zirconia abutment with a friction-fitted titanium insert showed fractures generated from the contact area between zirconia and the screw head. The zirconia abutment with the bonded titanium insert showed a separation between the two parts. Our results demonstrated a difference between the modes of failure in three types of zirconia abutment. However, the present study demonstrated that zirconia abutments with titanium inserts had a similar failure mode to different fracture surface heights, which depended on the height of the titanium inserts.

Clinically, the deeper the abutment structure damaged may make the clinicians more difficult to manage, increasing the complexity of complications. In a systematic review, Gou et al. [41] showed that, when fractures occur in external connection and two-piece internal connection zirconia abutments, the fragments are easily removed because the fractures occur above the implant shoulder. In contrast, when the one-piece internal connection zirconia abutment fractures, it may be difficult to retrieve the zirconia fragments. In most cases, zirconia fragments can be removed with an extraction bolt; however, removing the fragments would become more challenging when the extraction bolt also fractured and blocked the access hole [42]. The removal of fractured components may cause irreversible damage to the implant platform and internal structure, and if catastrophically damaged, the implant fixture may need to be surgically removed or replaced. Therefore, the titanium inserts might keep the fracture surface away from the implant platform and is beneficial to the clinicians to replace implant prostheses. In this study, there was no catastrophic damage or fracture of the abutment screws or implants found, as is consistent with the results of a study conducted by Mitsias et al. [17]. This mode of failure is quite different from titanium abutments. Yilmaz et al. [43] investigated five different titanium abutments for the load to failure, with four showing retentive screw fractures. Only one abutment did not show any components to fracture, but, eventually, bending of the screw beyond the plastic range did occur.

The limitations of this study require careful interpretation to explain the clinical implications correctly. The first is the use of static rather than cyclic load in this study. However, this static load test can be regarded as a preliminary study; future fatigue load projects can be designed based upon the mean failure load in this study. Second, this study uses only internal hexagonal connections and standard diameter implants with one implant system. The results may not be applicable to other implant systems. Lastly, the small number of samples in each group is also a limitation to be considered in this study. Therefore, additional clinical studies are needed to determine various zirconia abutments’ clinical performance and provide guidelines for clinical use.

## 5. Conclusions

Within the limitations of this in vitro study, the following conclusions were drawn:

1. There was a marginally significant difference in fracture resistance between the three groups, with the ASC zirconia abutment tending to have a higher fracture resistance than the full zirconia abutment.
2. The presence or absence of a titanium insert affects the modes of failure in zirconia abutments. When there is a titanium reinforcement, the abutment presents a relatively horizontal fracture surface; if not, it exhibits an oblique fracture. Furthermore, the titanium insert keeps the buccal fracture surface of the zirconia abutment away from the implant platform, thereby protecting the implant–abutment connection.

## Figures and Tables

**Table 1:** Zirconia abutments evaluated.

Material	Abutment Composition	Abutment/Implant Platform Interface	Manufacturer
OEM NobelProcera CAD/CAM Zirconia Abutment	Zirconia	Zirconia/Titanium	Nobel Biocare, Yorba Linda, Calif
OEM NobelProcera CAD/CAM ASC Abutment	Zirconia + Ti insert (friction fit)	Titanium/Titanium	Nobel Biocare, Yorba Linda, Calif
Aftermarket CAD/CAM zirconia abutment on Ti insert	Zirconia + Ti insert (bonded)	Titanium/Titanium	JingGang, Tainan, Taiwan

**Table 2:** Mean fracture resistance of zirconia abutments.

Mean Fracture Resistance (N)			
Group	N	Mean	SD
NobelProcera CAD/CAM zirconia abutment	5	252.37	82.79
NobelProcera CAD/CAM ASC Abutment	5	384.62	45.24
Aftermarket CAD/CAM zirconia abutment on Ti insert	5	361.83	90.31

**Table 3:** Modes of failure and mean distance from fracture surface to implant platform of zirconia abutments.

Mean (SD) Distance from Fracture Surface to Implant Platform					
Group	*n*	Height of Ti Inserts	Mid-Buccal	Mid-Palatal	Buccal-Lingual Discrepancy
OEM NobelProcera CAD/CAM zirconia abutment	5		0 mm	5.11 ± 1.47 mm	5.11 ± 1.47 mm
OEM NobelProcera CAD/CAM ASC Abutment	5	1 mm	3.52 ± 0.44 mm	3.82 ± 0.74 mm	0.30 ± 0.31 mm
Aftermarket CAD/CAM zirconia abutment on Ti insert	5	2 mm	4.12 ± 0.13 mm	5.18 ± 0.18 mm	1.06 ± 0.04 mm

## Data Availability

Correspondence and requests for materials should be addressed to Aaron Yu-Jen Wu.
